# Genomic Analysis of *Salmonella enterica* Serovar Typhimurium DT160 Associated with a 14-Year Outbreak, New Zealand, 1998–2012

**DOI:** 10.3201/eid2306.161934

**Published:** 2017-06

**Authors:** Samuel J. Bloomfield, Jackie Benschop, Patrick J. Biggs, Jonathan C. Marshall, David T.S. Hayman, Philip E. Carter, Anne C. Midwinter, Alison E. Mather, Nigel P. French

**Affiliations:** Athor affiliations: Massey University, Palmerston North, New Zealand (S.J. Bloomfield, J. Benschop, P.J. Biggs, J.C. Marshall, D.T.S. Hayman, A.C. Midwinter, N.P. French);; Institute of Environmental Science and Research, Wellington, New Zealand (P.E. Carter);; University of Cambridge, Cambridge, UK (A.E. Mather)

**Keywords:** Salmonella, Salmonella enterica, serovar Typhimurium DT160, definitive type 160, genomics, epidemiology, molecular evolution, outbreak, New Zealand, origin, transmission, multi-host pathogen, humans, wild birds, poultry, bovids, bacteria, gastroenteritis, enteric infections

## Abstract

During 1998–2012, an extended outbreak of *Salmonella enterica* serovar Typhimurium definitive type 160 (DT160) affected >3,000 humans and killed wild birds in New Zealand. However, the relationship between DT160 within these 2 host groups and the origin of the outbreak are unknown. Whole-genome sequencing was used to compare 109 *Salmonella* Typhimurium DT160 isolates from sources throughout New Zealand. We provide evidence that DT160 was introduced into New Zealand around 1997 and rapidly propagated throughout the country, becoming more genetically diverse over time. The genetic heterogeneity was evenly distributed across multiple predicted functional protein groups, and we found no evidence of host group differentiation between isolates collected from human, poultry, bovid, and wild bird sources, indicating ongoing transmission between these host groups. Our findings demonstrate how a comparative genomic approach can be used to gain insight into outbreaks, disease transmission, and the evolution of a multihost pathogen after a probable point-source introduction.

Nontyphoidal serovars of *Salmonella enterica* subsp. *enterica*, which cause salmonellosis, are responsible for an estimated 93.8 million illnesses and 155,000 deaths among humans worldwide each year (*1*). In New Zealand, these serovars are the second largest cause of bacterial gastroenteritis, annually causing 21 cases per 100,000 population (*2*). Nontyphoidal *Salmonella* spp. strains vary in host specificity and are usually transmitted to humans via direct contact or consumption of foods originating from animals (*3*,*4*). In New Zealand, salmonellosis incidence among humans peaks in the warm summer months, probably in association with increased multiplication of *Salmonella* in animal and food sources and with increased participation in higher risk outdoor activities (e.g., activities that increase contact with wild-life) (*5*). Climate change is expected to increase summer temperatures, potentially increasing salmonellosis incidence in New Zealand (*6*).

During 1998–2012, an extended outbreak of *Salmonella* Typhimurium definitive type 160 (DT160) occurred in New Zealand (*7*). During the outbreak, DT160 was the predominant *Salmonella* spp. subtype isolated from human salmonellosis patients and sick wild birds. DT160 was also isolated from other animals and the environment, but it was not the main *Salmonella* subtype isolated from these sources (*8*–*10*). DT160 has been isolated from animals and environments worldwide (*11*,*12*) and is usually associated with moribund birds (*13*,*14*). However, before the 1998–2012 outbreak, DT160 had not been reported in New Zealand. In 2009, an outbreak of DT160 involving humans and wild birds was reported in Tasmania, Australia (*15*); however, as with the outbreak in New Zealand, the relationship between DT160 within the bird and human host groups of Tasmania was unknown. We used genomic epidemiologic approaches to characterize the origin, evolution, and transmission of *Salmonella* Typhimurium DT160 in New Zealand.

## Methods

### Whole-Genome Sequencing

After stratifying the *Salmonella* strain collection at the Enteric Reference Laboratory of the Institute of Environmental Science and Research Ltd. (Wallaceville, New Zealand) by age and host, we randomly selected 35 human, 25 wild bird, 25 poultry, and 24 bovine DT160 isolates from 1998–2012. We extracted genomic DNA from these isolates using a QIAamp DNA Mini Kit (QIAGEN, Hilden, Germany) (*16*). New Zealand Genomics Limited (NZGL) at Massey Genome Service, Massey University, Palmerston North, New Zealand, performed whole-genome sequencing of the extracts. NZGL also prepared a library for each isolate by using a TruSeq DNA PCR-Free Library Preparation Kit (Illumina, Scorsby, Victoria, Australia) and sequenced the libraries by using MiSeq (Illumina, San Diego, CA, USA) as 2 × 250 bp paired-end runs (≈120–150 genome coverage). After sequencing and standard barcode demultiplexing, NZGL used FASTQ-MCF (*17*) to perform quality control procedures to remove any PhiX control library reads and adaptor sequences. The raw reads for the 109 DT160 isolates are available in the European Nucleotide Archive (http://www.ebi.ac.uk/ena; accession no. PRJEB18077).

### Genomic Assembly

Each isolate’s genome was assembled de novo. We used an in-house Perl script to trim reads at an error probability of 0.01 and generate random subsets of paired reads from 750,000 to 1.2 million paired reads in increments of 150,000, varying the average coverage. We assembled each of the random sets by using the de novo assembler Velvet version 1.1 (*18*) at a variety of k-mers (from 55 to 245) in increments of 10. De novo assembly resulted in multiple genome assemblies for each isolate. We ranked the metrics for each of 4 parameters (longest genome length, fewest number of contigs, largest N_50_ value, and longest contig length) in numeric order and calculated an overall equally summed ranking score for each assembly. We used the assemblies with the lowest total rank for further analyses. We used QUAST (*19*), a quality assessment tool for evaluating and comparing genome assemblies, to analyze the DT160 de novo assemblies and determine their GC content (i.e., the percentage of a DNA sequence made up of guanine and cytosine bases).

### Single-Nucleotide Polymorphism Identification

We used Snippy version 2.6 (https://github.com/tseemann/snippy) and kSNP version 3.0 (*20*) to identify core single-nucleotide polymorphisms (SNPs). Snippy is a pipeline that uses the Burrows-Wheelers Aligner (*21*) and SAMtools version 1.3.1 (*22*) to align reads from different isolates to a sequence and uses FreeBayes (*23*) to identify variants among the alignments. We used kSNP to analyze de novo assembled genomes, along with the reference genome, *S. enterica* serovar Typhimurium 14028S (GenBank accession no. NC_016856). We used an in-house Python script to determine the read coverage of all the SNPs identified via kSNP. We used Snippy to align reads from each isolate to the reference genome (GenBank accession no. NC_016856) before identifying SNPs. SNPs were accepted if they had a >10 read depth and a >90% consensus for each isolate. The position of the SNP on the reference genome was used to determine if both methods identified the SNP or if they were unique to the method (Technical Appendix). This method identified 793 core SNPs shared by the 109 New Zealand DT160 isolates.

### Global DT160 Strains

Using the genomic assembly and SNP identification methods as we described, we compared 2 DT160 strains from the United Kingdom with the 109 DT160 isolates from New Zealand: 1,521 core SNPs were identified. We downloaded the UK strains, which were previously published by Petrovska et al. (*24*), from the European Nucleotide Archive (accession nos. ERS015626 and ERS015627).

### Phylogenetic Inference and Distances

We used RAxML version 8.2.4 (*25*) to construct a maximum-likelihood tree based on the 793 core SNPs of the 109 DT160 isolates; we used EvolView version 2 (*26*) to visualize and edit the tree. We used SplitsTree (*27*) to form a NeighborNet tree of the 109 New Zealand DT160 isolates based on the 793 core SNPs that they share and to compare the New Zealand and UK isolates based on the 1,521 core SNPs that they share. We used MEGA6 (*28*) and the maximum composite likelihood model (29) to predict the pairwise distance between the 109 New Zealand DT160 isolates, based on the 793 core SNPs they share, and the 109 New Zealand and 2 UK isolates, based on the 1,521 core SNPs that they share.

### Phylogenetic Analysis

We used an in-house Perl script to split the 793 codons into 5 groups: those associated with the first, second, or third codon; those contained in overlapping coding regions; and those found in intergenic regions. We also used the in-house Perl script to determine whether the SNPs were synonymous or nonsynonymous. We then exported the partitioned SNPs into BEAUti to create an XML file for BEAST 1.8.3 (*30*). 

To allow for variation in base substitution among codon positions, we used separate Hasegawa Kishino Yano models to estimate the 5 SNP groups (*31*); to allow for and estimate changes in the effective population size, we used the Gaussian Markov random field Bayesian skyride model (*32*); to allow for variation in mutation rates among lineages, we used an uncorrelated relaxed molecular clock (*33*), which was calibrated by the tip dates. We ran the XML file in BEAST for 40 million steps a total of 3 times with different starting seeds before using LogCombiner (http://beast.bio.ed.ac.uk/LogCombiner) to combine the runs with a 10% burn-in. To visualize the results and the relative change in effective population size, we used Tracer version 1.6 (*34*).

To determine the mutation rate for the DT160 genome, we multiplied the mutation rate estimated by BEAST by the number of analyzed core SNPs (793 bp) and then divided the product by the mean genome size of the analyzed isolates (4,884,485 bp). We used the discrete phylogeographic model (*35*) to predict ancestral migrations between host groups over the course of the outbreak.

### Protein Coding Gene Analysis

We used Prokka (*36*) to annotate de novo assembled genomes, and we used Roary (*37*) to cluster proteins and identify those that were found only in a subset of isolates and those that differed in length between the isolates. We used ClustalW version 2.1 (*38*) to align amino acid sequences, and we used an in-house Perl script to determine if these alignments contained mismatches. The nucleotide sequence of all proteins that differed were extracted from the assembled genomes, along with 500-bp flanks on either side of the sequence, by using an in-house Perl script. We could not obtain 500-bp flanks for some genes because they were located at the end of contigs. For those genes, the flank was cut short, but their length was annotated. We extracted flanks to help with read alignment. This extraction left a pool of nucleotide sequences from each isolate, for every protein that potentially differed in sequence. For each protein, we extracted all nucleotide variants from the pool by using an in-house Perl script. We used SRST2 version 2, a read mapping–based tool (*39*), to align reads from each isolate to the sequence variants, and we used SAMtools version 1.3.1 (*22*) to form a consensus sequence from the aligned reads. We set the consensus cutoff at a read depth of >8 and a consensus of >80%. The flanks were removed from the consensus sequences, and the sequence variants were translated into amino acid sequences by using an in-house Perl script. We identified protein differences by comparing the amino acid sequences from each isolate and combined the differences with the nonsynonymous SNPs identified by SNP analysis. The position of nonsynonymous SNPs within proteins was used to prevent repeats.

We used the Clusters of Orthologous Groups of proteins (COGs) database (*40*) to predict protein functions. For each functional group, we calculated the proportion of proteins that differed in sequence, and we used a Fisher exact test, computed via Monte Carlo Markov Chains of ≈10^9^ iterations, to determine if there were any differences between these proportions.

We used an in-house Perl script to form a presence–absence matrix of all the protein differences. We used Primer-E version 6 (*41*) to predict the Euclidian distance between the isolates based on the presence–absence matrix. The centroid is the arithmetic mean for a group of data points in an n-dimensional space. To assess differences in centroids among isolates collected from different sources or time periods, we applied PERMANOVA (http://www.primer-e.com/permanova.htm). To assess differences in dispersions between different groups, we computed dispersions (z-values) by using PermDisp (*42*) and then modeled them using a regression model with date of collection and source as the explanatory variables.

### Scripts

The in-house scripts used for genomic analyses in this study were specifically designed for this dataset. The scripts are available from GitHub (https://github.com/samuelbloomfield/Scripts-for-genomic-analyses).

## Results

**Figure 1:**
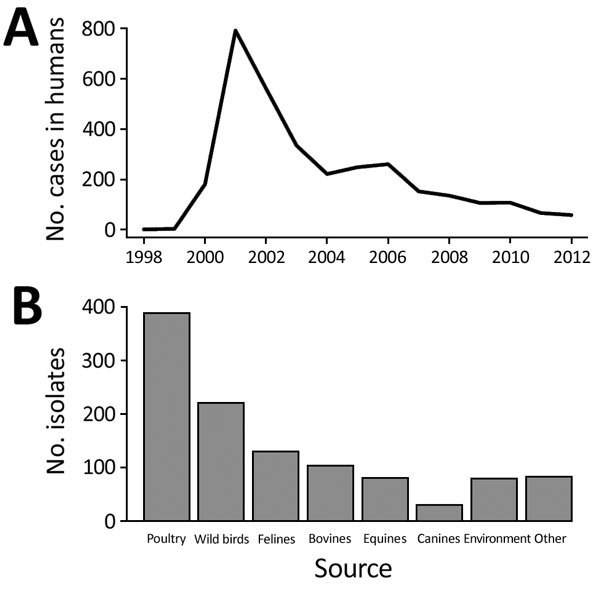
Number of *Salmonella enterica* serovar Typhimurium DT160 cases and isolates reported during an outbreak in New Zealand, 1998–2012. A) Cases in humans (*8*,*9*). B) Isolates from nonhuman sources (*8*,*10*).

During the 1998–2012 human outbreak of *Salmonella* Typhimurium DT160 in New Zealand, disease incidence displayed a typical epidemic curve: prevalence increased from 1999 to 2000, before peaking at 791 cases in 2001, and then slowly decreased from 2002 through 2012 (Figure 1). At the same time, numerous isolates were reported from nonhuman hosts (wild birds, poultry, bovids), and disease incidence among these host groups displayed epidemic curves similar to those for humans (Technical Appendix).

### Genomic DT160 Comparison

The genomes we assembled were 4.8–4.9 Mb in length and had a GC content of 52.11%–52.16% (reference value for *S. enterica* 50%–53%) (*43*). We identified 793 core SNPs shared by the 109 DT160 isolates from New Zealand.

### DT160 Introduction Date

Ancestral date reconstruction analysis predicted that the 109 New Zealand DT160 isolates shared a date of common ancestor in approximately August 1997 (95% highest posterior density interval June 1996–August 1998). Comparative analysis indicated that the 2 DT160 isolates collected from the United Kingdom were genetically distinct from the 109 New Zealand DT160 isolates (Technical Appendix). The average pairwise SNP distance between the 2 UK DT160 isolates and the New Zealand isolates was 0.0287, compared with an average pairwise distance of 0.0151 between New Zealand isolates.

In New Zealand, DT160 was first reported in Christchurch in 1998 from a human with salmonellosis (*44*) (an isolate from this case was included as part of this study). The New Zealand DT160 isolates we analyzed were estimated to share a common ancestor 0–2 years before this case and were distinct from the UK isolates analyzed, suggesting that DT160 was probably introduced into New Zealand as a single incursion within this time period. However, worldwide comparative studies are required to track DT160 migration and validate this hypothesis.

### DT160 Evolution

**Figure 2:**
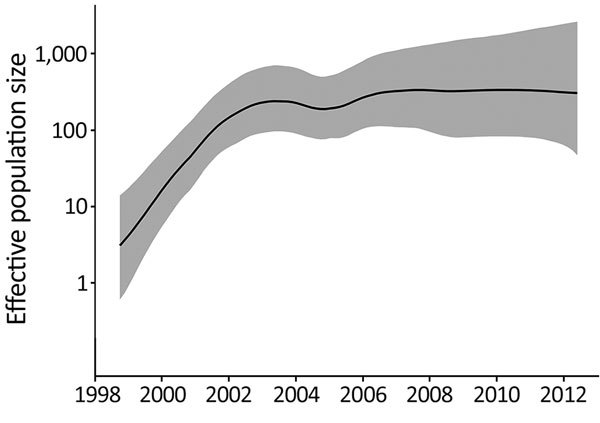
Relative effective population size (log scale) of *Salmonella enterica* serovar Typhimurium DT160 during an outbreak in New Zealand, 1998–2012. Population parameters were estimated using the Gaussian Markov random field Bayesian skyride model. The black line represents the median effective population size estimate; gray shading represents the 95% highest posterior density interval.

**Figure 3:**
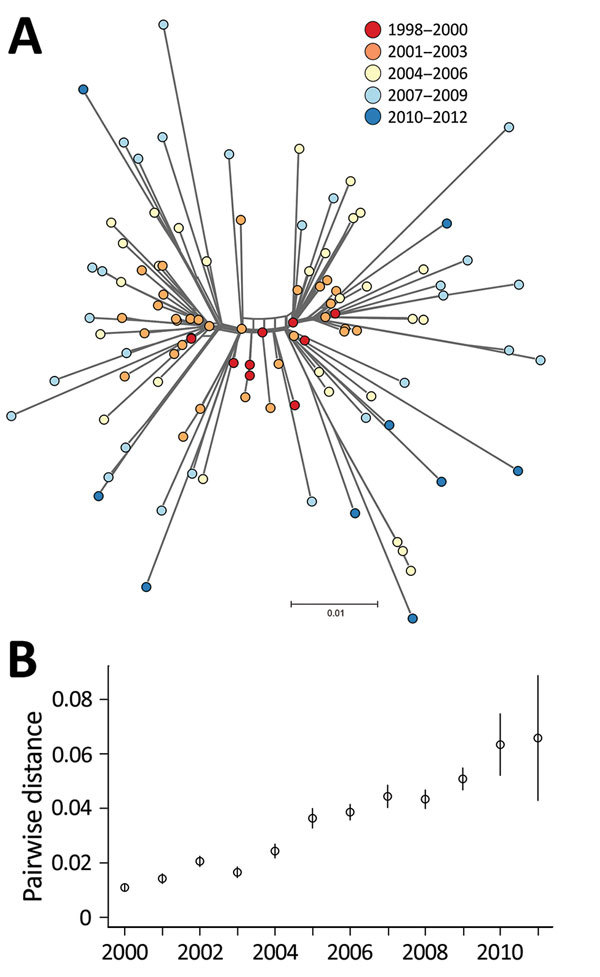
A) NeighborNet tree of 109 *Salmonella enterica* serovar Typhimurium DT160 isolates collected during an outbreak in New Zealand, 1998–2012. The tree was based on 793 core single-nucleotide polymorphisms. Colors indicate date of isolate collection. The scale bar represents the number of nucleotide substitutions per site. B) Scatterplot of the mean pairwise distance of 106 DT160 isolates from 2000–2011. Error bars represent 95% CIs.

Our phylogenetic analysis also predicted that the 109 DT160 isolates mutated at a rate of 3.3–4.3 × 10^−7^ substitutions/site/year (95% highest posterior density interval) and that the effective population size for DT160 increased from 1998 to 2003 (Figure 2). Over the course of the outbreak, DT160 also increased in genetic diversity (Figure 3).

The mutation rate estimated for the DT160 outbreak is similar to rates reported by Mather et al. (*45*) for an outbreak of *Salmonella* Typhimurium DT104 in Scotland during 1990–2012 and by Okoro et al. (*46*) for invasive *Salmonella* Typhimurium strains in sub-Saharan Africa. The similarity of these mutation rates suggests consistency between outbreaks caused by *S. enterica* serovar Typhimurium and has implications for modeling the evolution of future outbreaks caused by this serovar.

In bacteriology, the effective population size is the number of bacteria that contribute to the next generation. The increase in the DT160 effective population size during 1998–2003 coincided with an increased prevalence of DT160 among human and nonhuman hosts during this time. However, the subsequent levelling-off of the effective DT160 population size is probably an artifact because we calculated the effective population size from the timing of coalescent events for randomly sampled bacteria (*32*), and as the outbreak proceeded, fewer coalescent points were available for estimation.

Overall, our phylogenetic analyses suggest that the DT160 population increased dramatically in the first few years following introduction. As the DT160 population increased, it acquired multiple SNPs, resulting in a progressive increase in diversity over time.

### DT160 Sources

**Figure 4:**
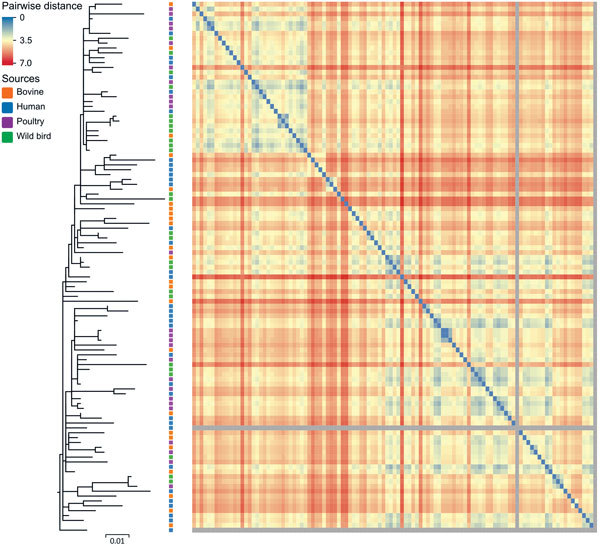
Maximum-likelihood tree of 109 *Salmonella enterica* serovar Typhimurium DT160 isolates collected during an outbreak in New Zealand, 1998–2012. The tree was based on 793 core single-nucleotide polymorphisms. Colored squares to the right of the branches indicate the source of isolates. The scale bar represents number of nucleotide substitutions per site. The heat map represents the Euclidean pairwise distance between isolates (based on the presence of 684 protein differences). Isolates that shared a small number of protein differences contained small Euclidean distances and are closer to blue in color on the heat map; isolates that shared a large number of protein differences contained large Euclidean distances and are closer to red in color. The gray squares represent the 2 outliers missing a large number of genes. The diagonal array of blue squares represents the pairwise distance for the same isolates.

PFGE (pulsed-field gel electrophoresis) was previously used to compare New Zealand DT160 isolates from humans, poultry, and wild birds (S. Omar, master’s thesis, 2011; http://mro.massey.ac.nz/handle/10179/2681?show=full); however, PFGE could not distinguish DT160 from the separate sources. In our study, we were able to use whole-genome sequencing to distinguish DT160 at the isolate level. However, we did not find any distinct DT160 clades associated with any one source (Figure 4).

Identifying the source of a salmonellosis outbreak can be difficult because multiple potential sources must be considered (*47*). Probable sources of *Salmonella* can be identified by comparing isolates from infected humans with those from other human, nonhuman, and environmental sources (*48*). We did not find distinct DT160 clades associated with any 1 source, suggesting that after its introduction into New Zealand, DT160 was transmitted between multiple hosts, resulting in large epidemics among humans and wild birds. Our results also suggest that humans obtained DT160 from multiple sources over the course of the outbreak. This finding is consistent with that in a case-control study performed by Thornley et al. (*44*), which found that human DT160 cases were associated with multiple risk factors involving different sources: handling dead wild birds, contact with persons with diarrhea, and consumption of fast food.

### Ancestral Migration between Hosts

We used the discrete phylogeographic model to predict ancestral migration of DT160 between the animal and human host groups, similar to Mather et al. (*45*). However, we were unable to detect a signal that could not be attributed to different sampling fractions in the host groups (Technical Appendix). Therefore, an alternate method, larger sample size, or both are required to predict these ancestral migrations.

### Protein and Gene Analysis

Protein annotation identified 5,096 coding DNA sequences, of which 4,983 (98%) were found in all of the isolates, 108 (2%) were found in 95%–99% of isolates, and 3 (<1%) were found in 1%–5% of the isolates. Protein coding gene analysis also identified 477 nonsynonymous SNPs, of which 27 were nonsense mutations and 96 were INDELs (insertions/deletions). The nonsense SNPs and INDELs were responsible for 123 proteins that differed in length. Overall, we identified 684 differences in 604 protein sequences among the 109 DT160 isolates. We excluded 2 isolates from protein coding gene analysis because they were missing a large number of proteins (Technical Appendix).

**Figure 5:**
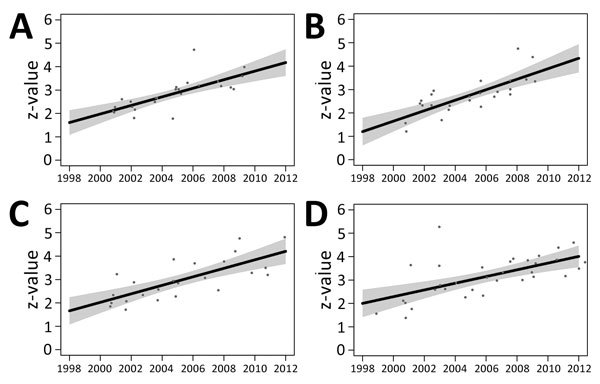
Scatter plots of year of collection versus z-values for 107 *Salmonella enterica* serovar Typhimurium DT160 isolates collected during an outbreak in New Zealand, 1998–2012. Of the 107 isolates, 25 were from poultry (A), 25 from wild birds (B), 24 from bovids (C), and 33 from humans (D). Black lines represent the regression equation; gray shading represents SE for this equation. Date of collection was significantly associated with z-values in this model (p<2^−16^). There was insufficient evidence to suggest that source was associated with z-values (p = 0.558), and the interaction between source and date of collection was not significant (p = 0.458).

By using PERMANOVA, we found that centroids based on the 684 protein differences were indistinguishable among groups of DT160 isolates collected from different sources and time periods (Technical Appendix). PERMANOVA's inability to distinguish centroids appears to be due to the fact that DT160 isolates radiated out from a point source. The z-value is the distance from an isolate to the centroid of a group of isolates; we calculated the z-value for 107 DT160 isolates on the basis of 684 protein differences. Our regression modeling results showed that the z-value was associated with the date, but not source, of collection (Figure 5).

The 684 protein differences shared by the DT160 isolates were associated with a large number of COG functional groups. The proportion of proteins that contained sequence differences differed between functional groups (p = 0.00002). The proportions varied from 0.06 to 0.18, although most were between 0.09 and 0.13 (Technical Appendix). In addition, our data were insufficient to model the effects of source or date of collection on the number of protein differences associated with each group (Technical Appendix).

Bacteria often adapt to new environments by altering (changing or losing) genes that are not essential for colonizing that environment (*49*). Gene loss can result in an increase in bacterial fitness, as fewer genes and processes need to be maintained within the bacteria (*50*). We identified multiple protein changes among the DT160 isolates, and these changes occurred in multiple COG functional groups as the epidemic progressed. However, we found no evidence of host group differentiation, suggesting that most of the evolution was due to random genetic drift rather than adaptive evolution.

## Discussion

Using genomic analysis, we described the evolution and emergence of *Salmonella* Typhimurium DT160 within New Zealand. Our results suggest that DT160 was introduced into New Zealand on a single occasion from 1996 through 1998, before propagating throughout the country and becoming more genetically diverse over time. In addition, we found that DT160 isolates collected from human, poultry, bovine, and wild bird sources were highly similar, indicating a large number of transmission episodes between these host groups.

Technical Appendix Additional materials and methods and results of various analyses, including maximum-likelihood and NeighborNet trees, for a study of the genomic analysis of *Salmonella enterica* serovar Typhimurium DT160 associated with a 14-year outbreak in New Zealand.
