# Supplementary material for: Genomic Analysis of Salmonella enterica Serovar Typhimurium DT160 Associated with a 14-Year Outbreak, New Zealand, 1998–2012
Source: Emerg Infect Dis. 2017 Jun;23(6):906–13. doi: 10.3201/eid2306.161934 (PMC5443446; doi:10.3201/eid2306.161934)
Supplement: Technical Appendix — Additional materials and methods and results of various analyses, including maximum-likelihood and NeighborNet trees, for a study of the genomic analysis of Salmonella enterica serovar Typhimurium DT160 associated with a 14-year outbreak in New Zealand. [file 16-1934-Techapp-s1.pdf]

Article DOI: https://dx.doi.org/10.3201/eid2306.161934

Genomic Analysis of *Salmonella enterica* Serovar Typhimurium DT160 Associated with a 14-Year Outbreak, New Zealand, 1998–2012

Technical Appendix

Sample Collection

From 1998–2012, *Salmonella enterica* serovar Typhimurium DT160 was isolated from humans and numerous animal and environmental sources in New Zealand. In this study, 35 human, 25 wild bird, 25 poultry and 24 bovine DT160 isolates were randomly selected from those isolates reported to the culture collection center at the Institute of Environmental Science and Research (ESR). The number of isolates reported in these host groups displayed similar epidemic curves, with an increase in prevalence from 1999–2000, before peaking in 2001 and slowly decreasing in prevalence from 2002–2012. (Technical Appendix Figure 1).

SNP Comparison

SNPs (single nucleotide polymorphisms) are single base pairs that differ between isolates. Two software programs were used to identify SNPs shared by the 109 DT160 isolates: Snippy (https://github.com/tseeman/snippy) and kSNP3 (*1*). Snippy was used to align reads from each isolate to a reference genome, in this case *S. enterica* serovar Typhimurium strain 14028s (NC_016856), and then to compare the alignment results and identify single base pairs that were found in all isolates but differed in sequence (core SNPs). kSNP was used to identify kmers of a fixed length that differed in one nucleotide between de novo-assembled genomes and NC_016856. kSNP identified 731 SNPs shared by the 109 DT160 isolates, while Snippy identified 771 SNPs (Technical Appendix Figure 2). 709 SNPs were identified by both methods, leaving 22 kSNP-unique and 62 Snippy-unique SNPs. The kSNP-unique SNPs mostly consisted of SNPs found on reads that did not align to the reference genome, while the Snippy-unique

SNPs mostly consisted of SNPs that were in close vicinity, unable to be picked up by kSNP as kmers of a fixed length would differ in more than one nucleotide. By using both methods a larger number of SNPs were identified than if a single method alone was used.

773 out of the 793 core SNPs shared by the 109 DT160 isolates were also located on the reference genome, NC_016856. The order of these SNPs on the reference genome identified several small clades associated with close clusters of SNPs (Technical Appendix Figure 3). However, most of the SNPs in these clusters were synonymous and unlikely to result from selection pressures. The order of these SNPs also identified the non-synonymous SNPs responsible for the formation of two distinct DT160 clades and the proteins they were located within: glycogen debranching enzyme (A), 2-dehydro-3-deoxyphosphooctonate aldolase (B), a YggT family protein (C), galactose-1-epimerase (D), uvrABC system protein B (E) and acrylyl-coA reductase (F). Many of these proteins are involved in carbohydrate metabolism, suggesting that the two DT160 clades may have distinct carbohydrate metabolism phenotypes.

Global DT160 Strains

Petrovska *et al*. (*2*) previously published the genomes of two DT160 isolates: ERS015626 that was isolated from a horse in 1998 and ERS015627 that was isolated from a bird in 1997. The raw reads from these isolates were downloaded from the European Nucleotide Archive (http://www.ebi.ac.uk/ena) and assembled de novo. kSNP and Snippy identified 1,521 core SNPs in total shared by these two isolates and the 109 New Zealand DT160 isolates analyzed. The average pairwise SNP distance between the two UK DT160 isolates and the New Zealand isolates was 0.0287, compared to an average pairwise distance between NZ isolates of 0.0151

The two DT160 isolates from the United Kingdom were genetically distinct from each other and from the 109 New Zealand DT160 isolates (Technical Appendix Figure 4). To our knowledge these were the only DT160 isolates published to date.

Protein Coding Gene Analysis

The 109 DT160 isolates shared 684 protein differences. Primer-E v6 (*3*) was used to predict the Euclidian distance matrix based on the presence of these protein differences.

Of the 684 proteins that differed in sequence, 546 (93%) contained a single protein difference (SNP, indel or presence), 53 (7%) contained two protein differences, and 5 (<1%) contained more than two (Technical Appendix Figure 5).

Two isolates were excluded from protein analyses as they lacked a large number of genes and were skewing the multi-dimensional scaling, functional plots and PermDisp calculations (Technical Appendix Figure 6). These outliers shared similar epidemiologic information: collected from human sources from 2004–2006. However, they were missing different sets of genes.

Multidimensional scaling helps visualize the amount of similarity or dissimilarity between data points. In multi-dimensional scaling, the centroid is the central point for a group of data points. PERMANOVA found that the centroids were indistinguishable between isolates collected from different sources or time periods (Table), as these isolates appeared to radiate out from a point source (Technical Appendix Figure 7).

The distance from the centroid to each isolate (z-value) is a measure of dispersion and equivalent to the accumulation of protein differences. The z-values were calculated using PermDisp (*4*) and were modeled using a regression model. The residuals for this model lacked normality (Technical Appendix Figure 8). To normalize the residuals, the z-values could have been transformed. However, with such a low p-value for the date of collection, this would not have changed the conclusions and would have made interpretation more difficult.

The 684 protein differences shared by the DT160 isolates were associated with a large number of functions. For each COG functional group, the proportion of proteins that differed in sequence was calculated (Technical Appendix Figure 9). Fisher exact test provided evidence that these proportions differed ($p = 0.0002$). However, there was little variation in the proportions (range: 0.07–0.17) and there were no outliers.

The mean proportion of proteins that differ in sequence for each functional group within each time period and source was calculated by dividing the proportion of proteins that differed in sequence among each source and time period in each functional group by the number of samples in each group (Technical Appendix Figures 10). Year of collection and source seemed to have a significant effect on the mean proportion of proteins that differ in sequence within each functional group: the proportion within each functional group tended to increase over time, and

certain functional groups (e.g., Extracellular structures (COG group W), Cell cycle control, cell division and chromosome partitioning (COG group D), Signal transduction mechanisms (COG group T), Lipid transport and metabolism (COG group I), and Cell motility (COG group N)) had higher proportions in the bovine and human host groups compared to the poultry and wild bird. However, the total number of protein differences within each functional group was smaller than the total number of samples (Technical Appendix Figure 11). Therefore, a regression model could not be used to model the effect of source and date of collection on the number of differences in each functional group, as a large number of isolates would have the same z-value.

Discrete Phylogeographic Model

The discrete phylogeographic model was designed to use phenotypic or molecular data to predict the ancestral migration of organisms from distinct geographies (*5*). However, the model has been applied to outbreaks to predict transmission between distinct host groups that share the same geography (*6*). Twenty-two datasets were formed from the 109 DT160 isolates and the 793 core SNPs they share, to determine if the discrete phylogeographic model was appropriate for investigating this outbreak. The real dataset consisted of the 109 isolates split into those from animal sources (n = 74) and those from human sources (n = 35) (real dataset). Ten datasets were formed by randomly assigning the 109 isolates as animal or human, while keeping the total number of animal and human isolates the same (datasets A-J). Eleven datasets were formed by randomly assigning one of the isolates as human, while assigning the rest as animal, before progressively assigning random isolates as human, until a range of data was formed with different numbers of human and animal isolates. Each dataset was exported into BEAUti to create an .xml file for BEAST 1.8.3 (*7*). For simplicity's sake, each dataset was given a separate Hasegawa Kishino Yano (HYK) substitution model (*8*) and strict molecular clock. The GMRF Bayesian skyride model (*9*) was used to allow for variation in the effective population size of each model and the discrete phylogeographic model (*5*) was used to predict the time spent in the animal and human host groups (Markov rewards) over the course of the outbreak, and the number of transmission between these host groups (Markov jumps). Each .xml file was run in BEAST for 10 million steps.

The discrete phylogeographic model predicted that DT160 spend most of the time in the animal host group, and that there was a larger amount of transmission from the animal to the human host group than the reciprocal. However, the same result was obtained when the isolates were randomly assigned as human or animal, but the sample proportions were kept the same (Technical Appendix Figures 12 and 13). In addition, the proportion of samples assigned as human had a significant effect on the Markov rewards and jumps (Technical Appendix Figures 14 and 15). This indicates that the results obtained from the discrete phylogeographic model are the result of an uneven sample size and not true migration events.

The proportion of samples that are human and Markov rewards share a step-like or sigmoid association (Technical Appendix Figure 14). This is due to the deep DT160 branches that are predominantly one source until the proportion of samples that are human meets a threshold (30%–40% of samples are human), where they suddenly all switch (Technical Appendix Figure 16). However, the relationship between the proportion of samples that are human and Markov jumps is more complex (Technical Appendix Figure 15). As the proportion of samples that are human increases, the number of human branches increases, but the ancestral branches remain animal, resulting in an increase in the number of animal-to-human Markov jumps. There are no human-to-animal Markov jumps up until the threshold, as there are no ancestral branches that are human. However, after the human proportion threshold is meet, the ancestral branches switch to human, resulting in no animal-to-human Markov jumps and a large number of human-to-animal Markov jumps that decrease as the human sample proportion increases and the number of animal tips decrease. If there were no deep branches or coalescent events, we would expect the correlation between the human proportion and Markov rewards to be more linear. In addition, we would expect there to be a positive linear relationship between the human proportion and the number of each Markov jump up to the threshold and a negative linear relationship afterwards.

References

1. Gardner SN, Slezak T, Hall BG. kSNP3.0: SNP detection and phylogenetic analysis of genomes without genome alignment or reference genome. Bioinformatics. 2015;31:2877–8. PubMed http://dx.doi.org/10.1093/bioinformatics/btv271

2. Petrovska L, Mather AE, AbuOun M, Branchu P, Harris SR, Connor T, et al. Microevolution of monophasic *Salmonella* Typhimurium during epidemic, United Kingdom, 2005–2010. Emerg Infect Dis. 2016;22:617–24. 10.3201/eid2204.150531 PubMed http://dx.doi.org/10.3201/eid2204.150531

3. Clark KR, Gorle RN. PRIMER v6: user manual/tutorial. Plymouth (UK): PRIMER-E; 2006. p. 296.

4. Anderson MJ. Distance-based tests for homogeneity of multivariate dispersions. Biometrics. 2006;62:245–53. PubMed http://dx.doi.org/10.1111/j.1541-0420.2005.00440.x

5. Lemey P, Rambaut A, Drummond AJ, Suchard MA. Bayesian phylogeography finds its roots. PLOS Comput Biol. 2009;5:e1000520. PubMed http://dx.doi.org/10.1371/journal.pcbi.1000520

6. Mather AE, Reid SWJ, Maskell DJ, Parkhill J, Fookes MC, Harris SR, et al. Distinguishable epidemics of multidrug-resistant *Salmonella* Typhimurium DT104 in different hosts. Science. 2013;341:1514–7. PubMed http://dx.doi.org/10.1126/science.1240578

7. Drummond AJ, Suchard MA, Xie D, Rambaut A. Bayesian phylogenetics with BEAUti and the BEAST 1.7. Mol Biol Evol. 2012;29:1969–73. PubMed http://dx.doi.org/10.1093/molbev/mss075

8. Hasegawa M, Kishino H, Yano T. Dating of the human-ape splitting by a molecular clock of mitochondrial DNA. J Mol Evol. 1985;22:160–74. PubMed http://dx.doi.org/10.1007/BF02101694

9. Minin VN, Bloomquist EW, Suchard MA. Smooth skyride through a rough skyline: Bayesian coalescent-based inference of population dynamics. Mol Biol Evol. 2008;25:1459–71. PubMed http://dx.doi.org/10.1093/molbev/msn090

10. Institute of Environmental Science and Research Ltd (ESR). ESR LabLink. Quarterly surveillance summaries for New Zealand, March 2000–March 2003 [cited 2016 Nov 25]. https://surv.esr.cri.nz/PDF_surveillance/Lablink/

11. Institute of Environmental Science and Research Ltd (ESR). Public Health Surveillance; Information for New Zealand public health action. 2003–2012 human *Salmonella* isolates [cited 2016 Nov 25]. https://surv.esr.cri.nz/enteric_reference/human_salmonella.php

12. Institute of Environmental Science and Research Ltd (ESR). Public Health Surveillance; Information for New Zealand public health action. Non-human *Salmonella* isolates, 2003–2012 [cited 2016 Nov 25. https://surv.esr.cri.nz/enteric_reference/nonhuman_salmonella.php

Technical Appendix Table. PERMANOVA (http://www.primer-e.com/permanova.htm) output for 107 *Salmonella enterica* serovar Typhimurium DT160 isolates, based on the presence of 684 protein differences and grouped by year of collection and source*

Coefficient	Df	SS	MSS	Pseudo-F	P(perm)	Unique perms
Year	4	42.26	10.57	1.143	0.121	998
Source	3	26.9	8.968	0.97	0.515	997
Year ×Source†	10	99.9	9.99	1.081	0.187	996
Residuals	89	822.8	9.245			
Total	106	1,002				

*Df, degrees of freedom; SS, sum of squares; MSS, mean sum of squares; Pseudo-F, F-value from the data; P(perm), proportion of permuted datasets whose F-value exceeds Pseudo-F; Unique perms, number of unique permutations.
†Coefficient interaction.

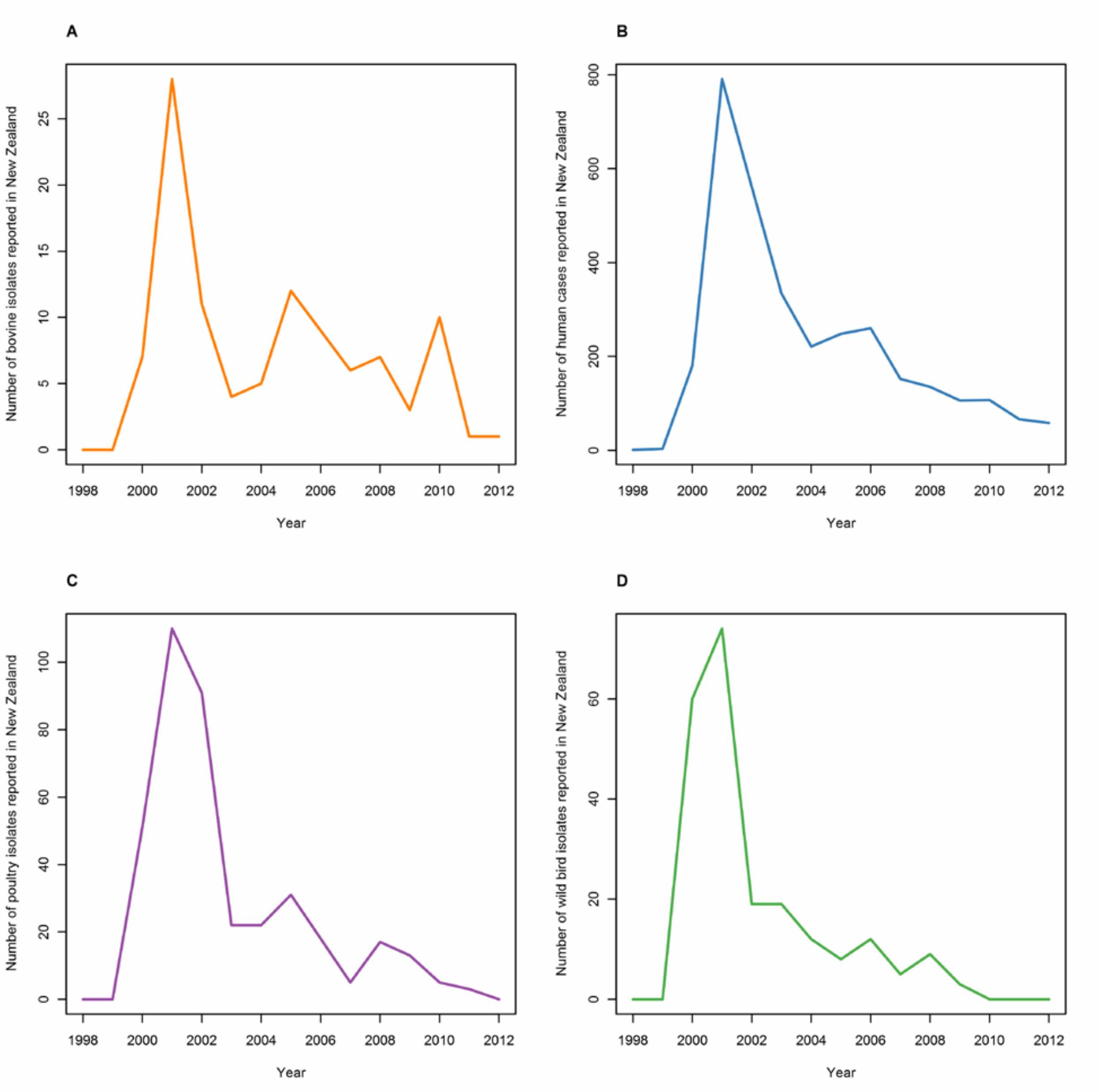

Technical Appendix Figure 1. Line graph of the number of bovine (A: orange), human (B: blue), poultry (C: purple) and wild bird (D: green) DT160 cases reported in New Zealand from 1998–2012 (*10*–*12*).

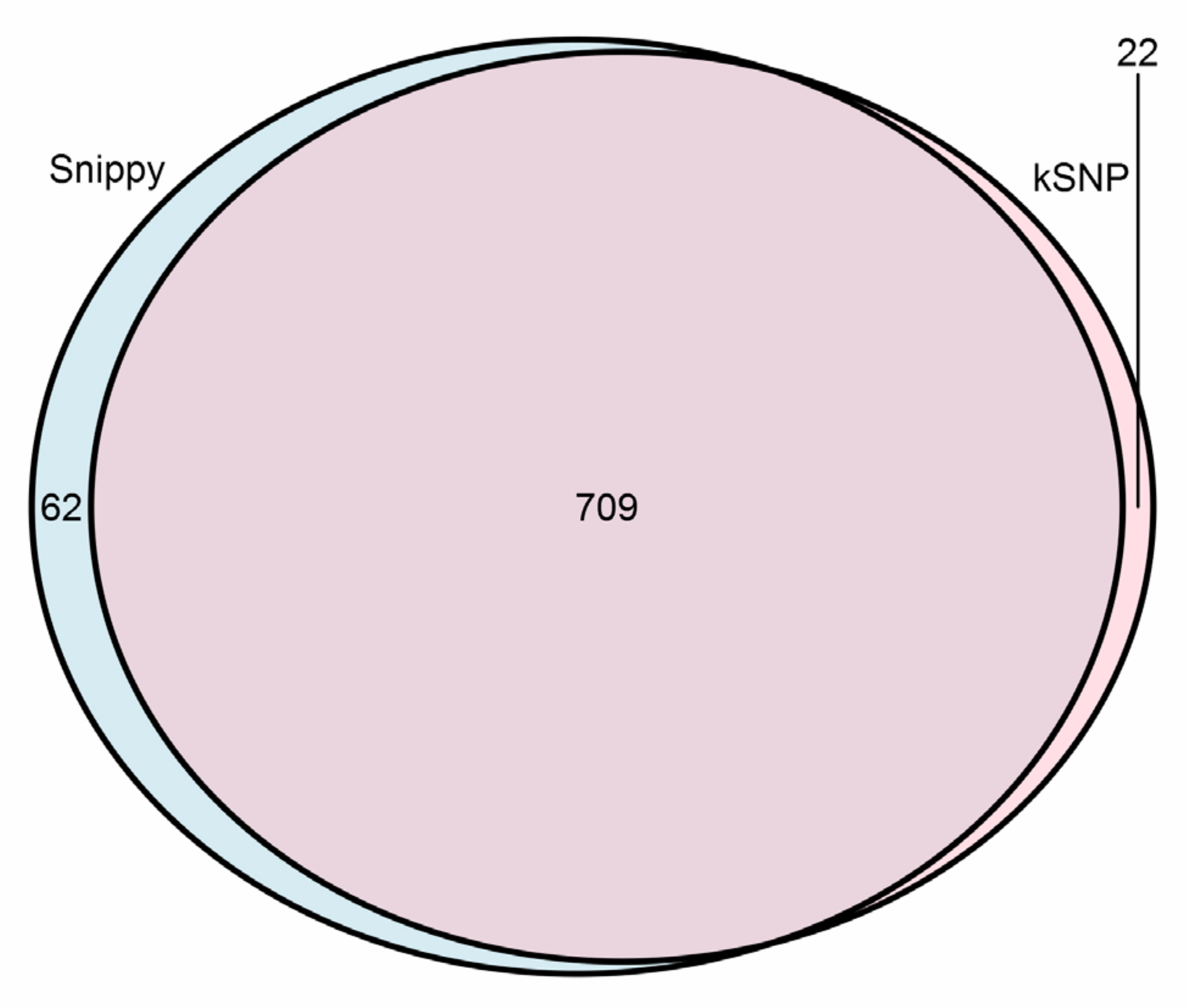

Technical Appendix Figure 2. Venn diagram of the number of unique and shared DT160 SNPs identified by Snippy and kSNP3.

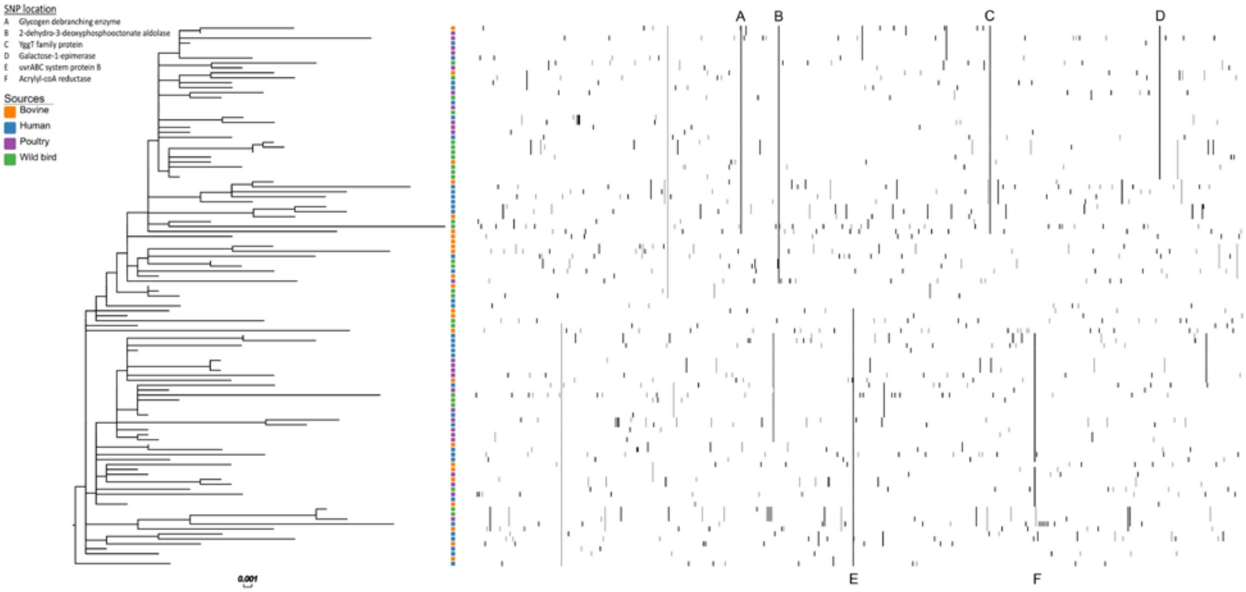

Technical Appendix Figure 3. Maximum likelihood tree of 109 DT160 isolates (based on 793 core SNPs). The scale bar represents the number of nucleotide substitutions per site. The colored squares represent the sources of the isolates. The presence-absence matrix represents the presence of the 773 core SNPs located on the reference genome, NC_016856. The SNPs were arranged in the order they appear on the reference genome. Black bars represent non-synonymous SNPs and gray bars represent synonymous SNPs. The non-synonymous SNPs responsible for the formation of the major DT160 clades were assigned a letter (A-F) and the proteins they are located within are outlined.

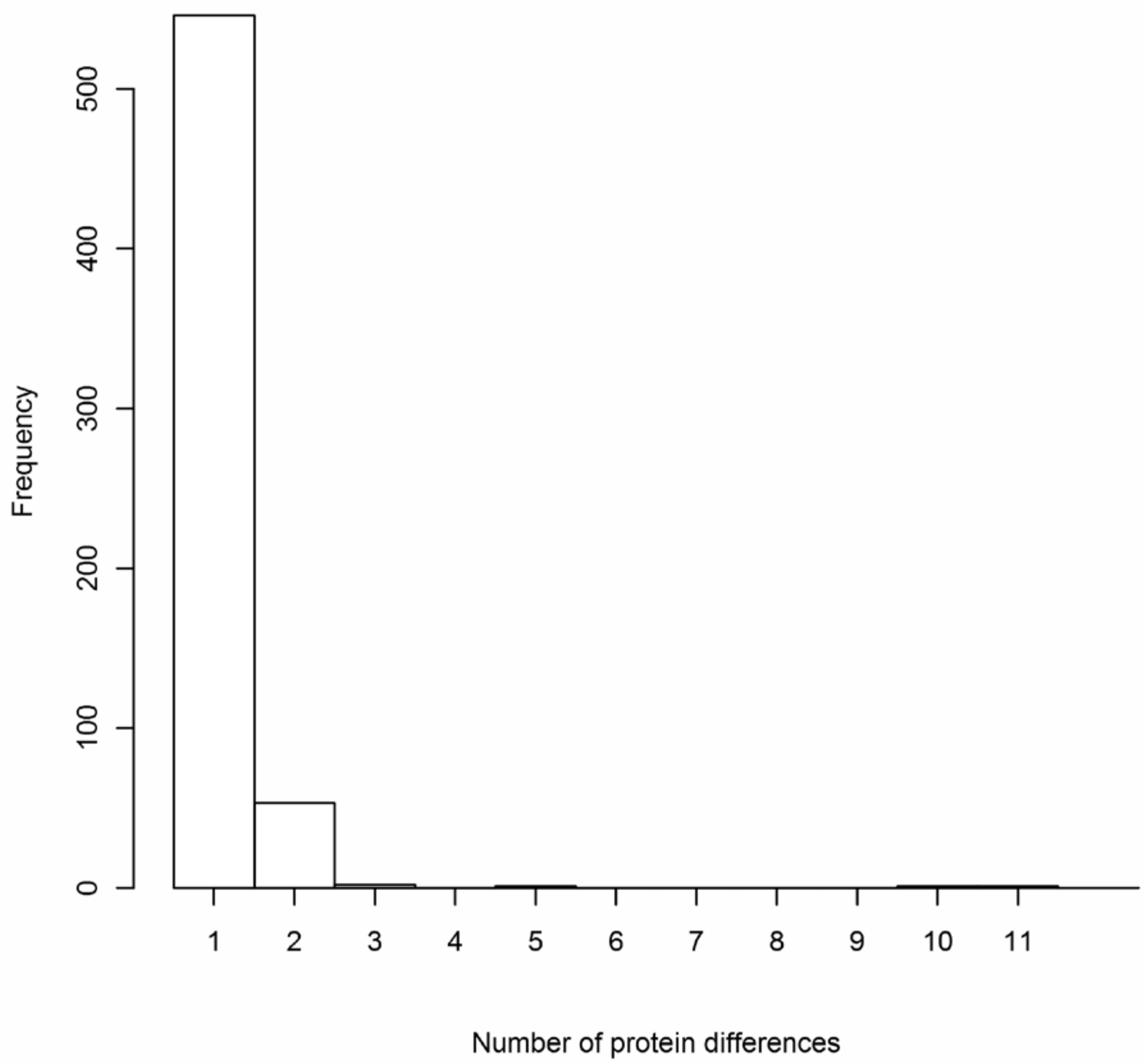

Technical Appendix Figure 4. Histogram of the number of protein differences found within the same protein sequence.

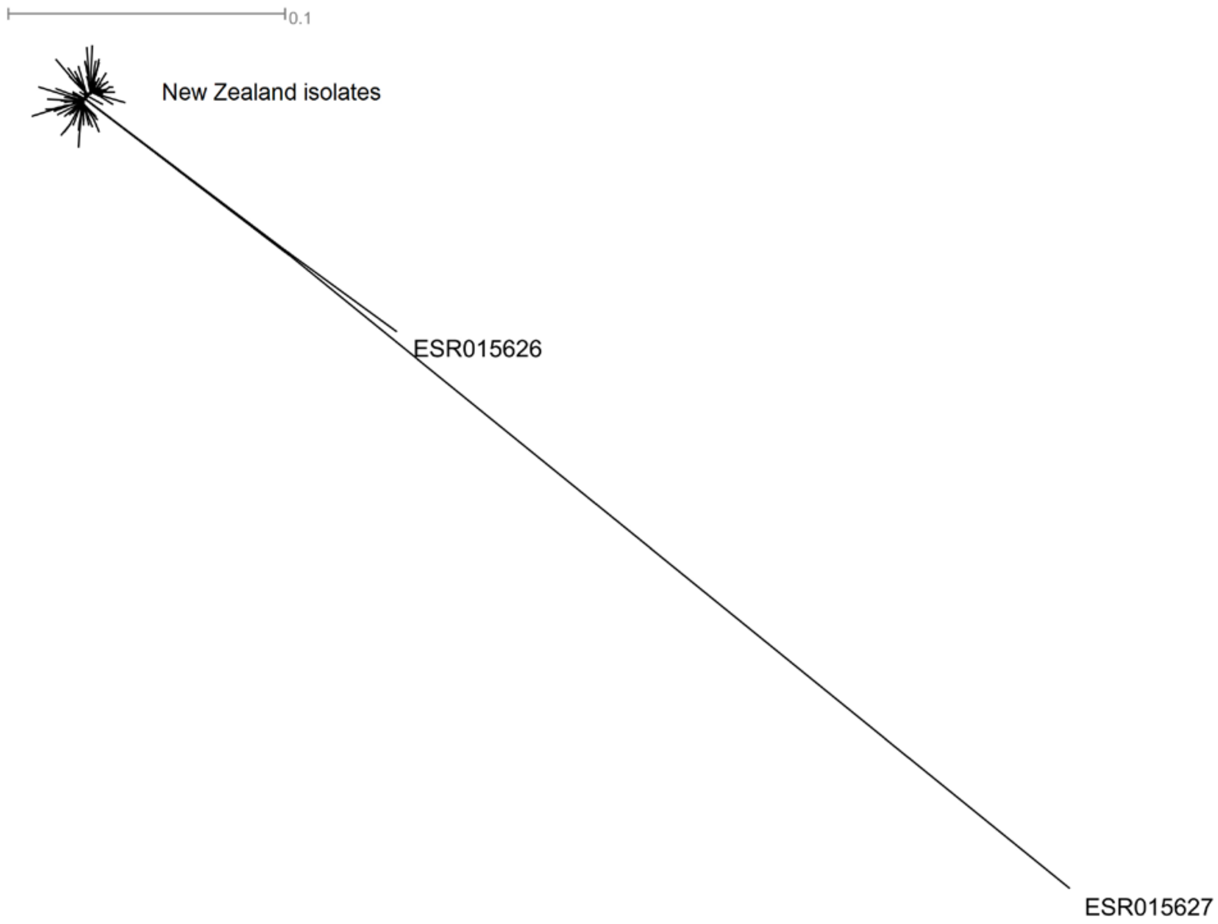

Technical Appendix Figure 5. NeighborNet tree of 111 DT160 isolates (based on 1,521 core SNPs): 109 from New Zealand and two from the United Kingdom (ERS015626 and ERS015627). The scale bar represents the number of nucleotide substitutions per site.

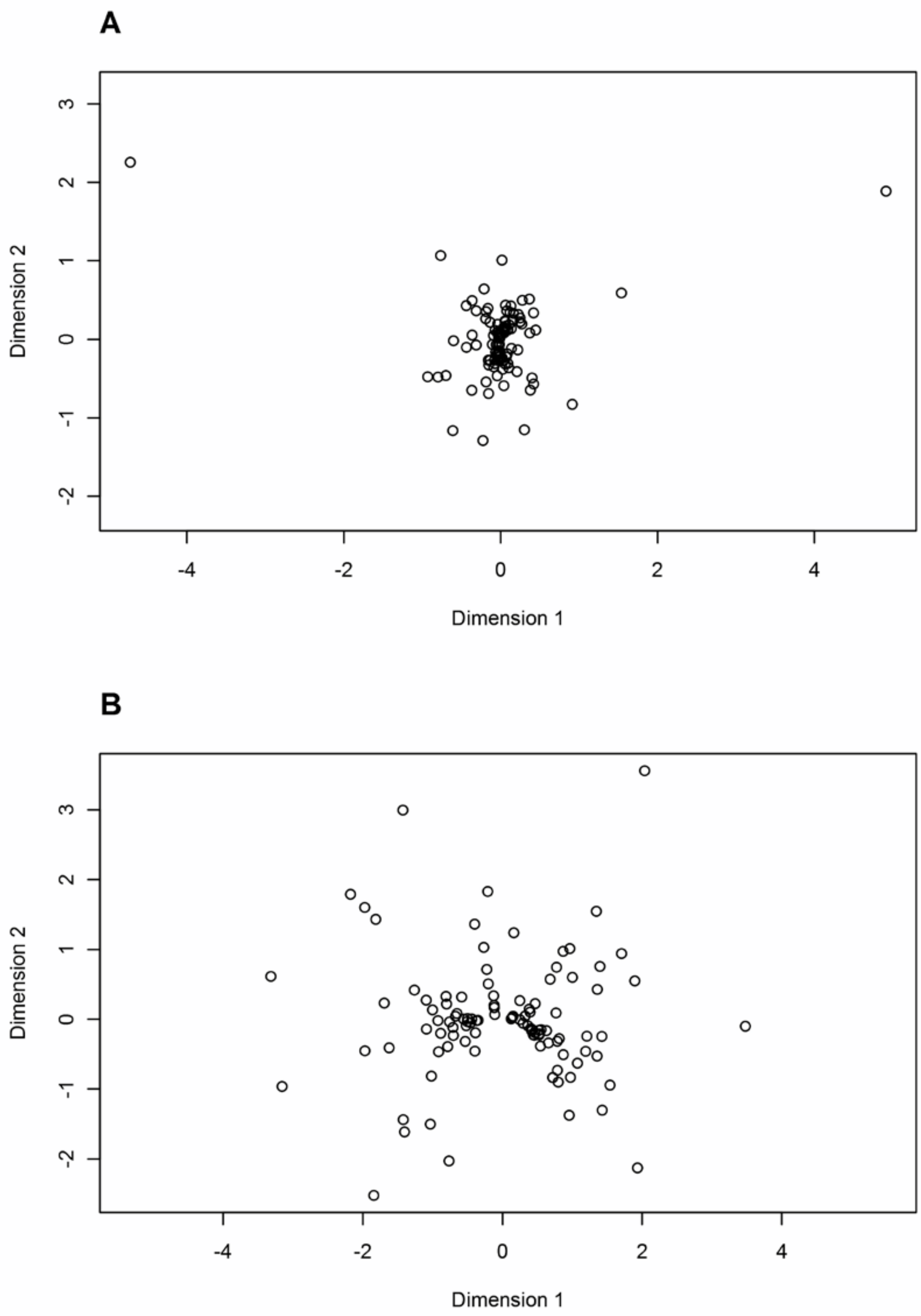

Technical Appendix Figure 6. Multi-dimensional scaling of 109 (A) and 107 (minus two outliers) (B) DT160 isolates based on the presence of 684 protein differences.

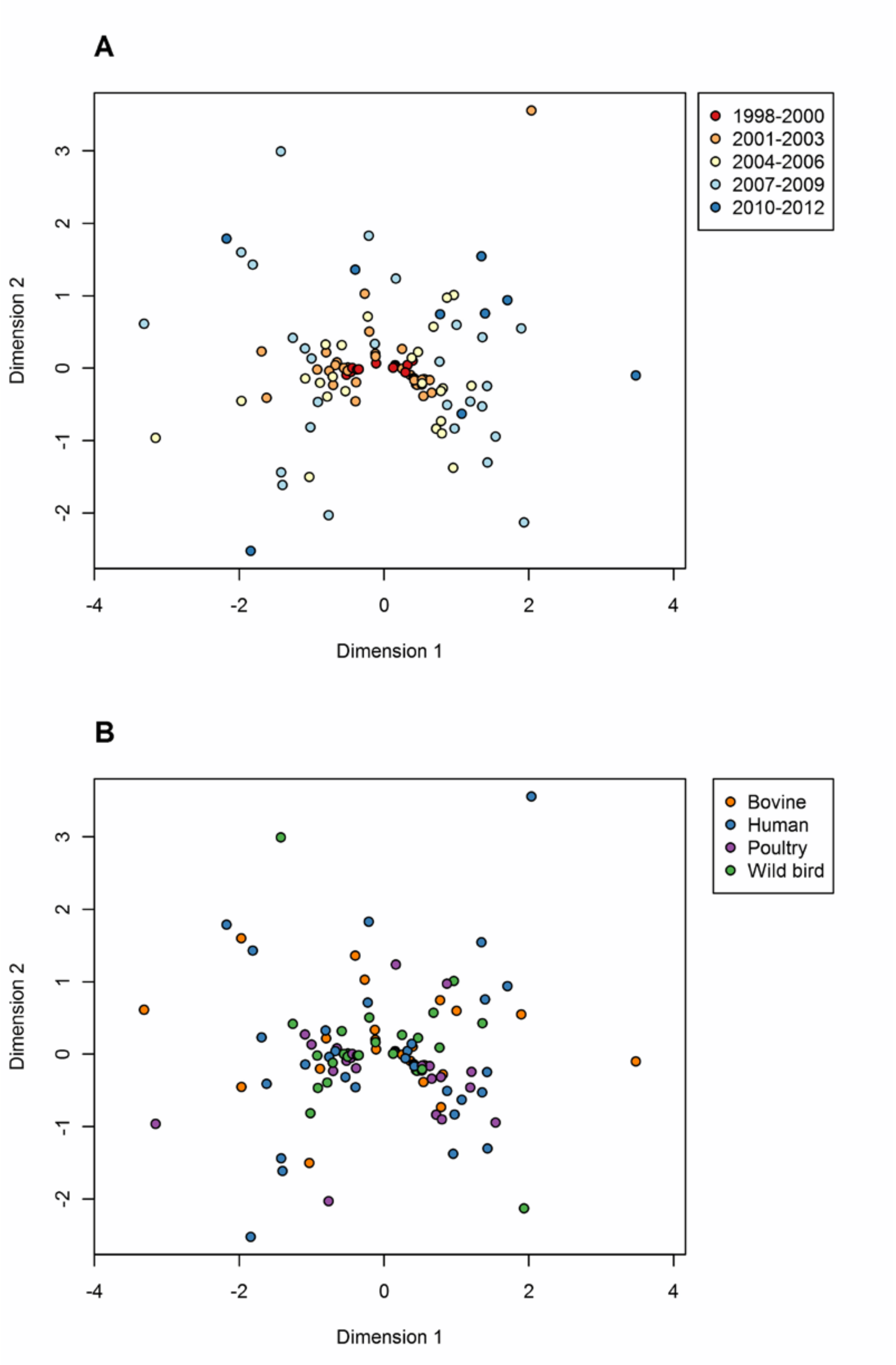

Technical Appendix Figure 7. Multi-dimensional scaling of 107 DT160 isolates, based on the presence of 684 protein differences and colored by date of collection (A) and source (B).

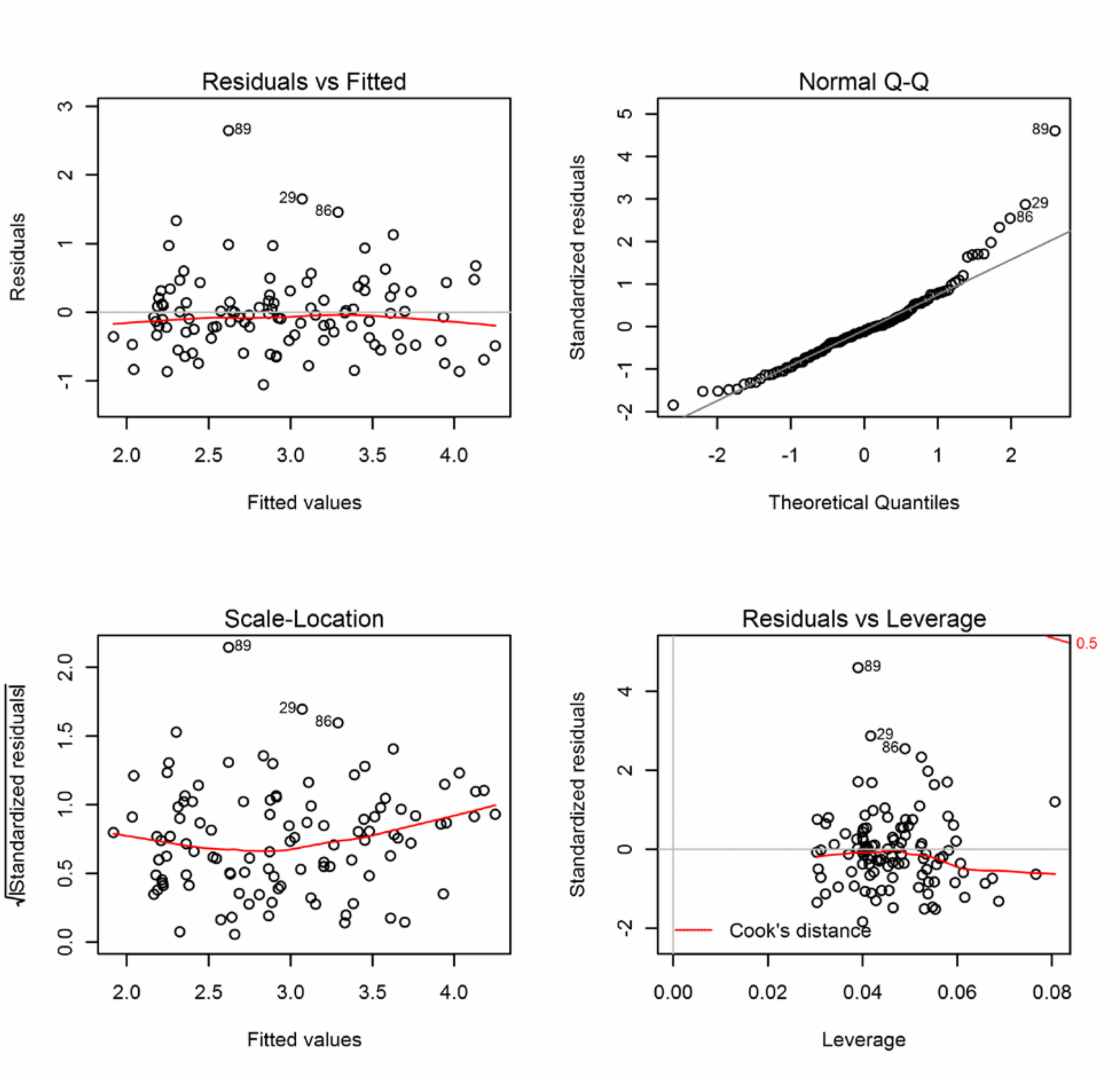

Technical Appendix Figure 8. Diagnostic plots of the regression model fitted to the z-values for 107 DT160 isolates.

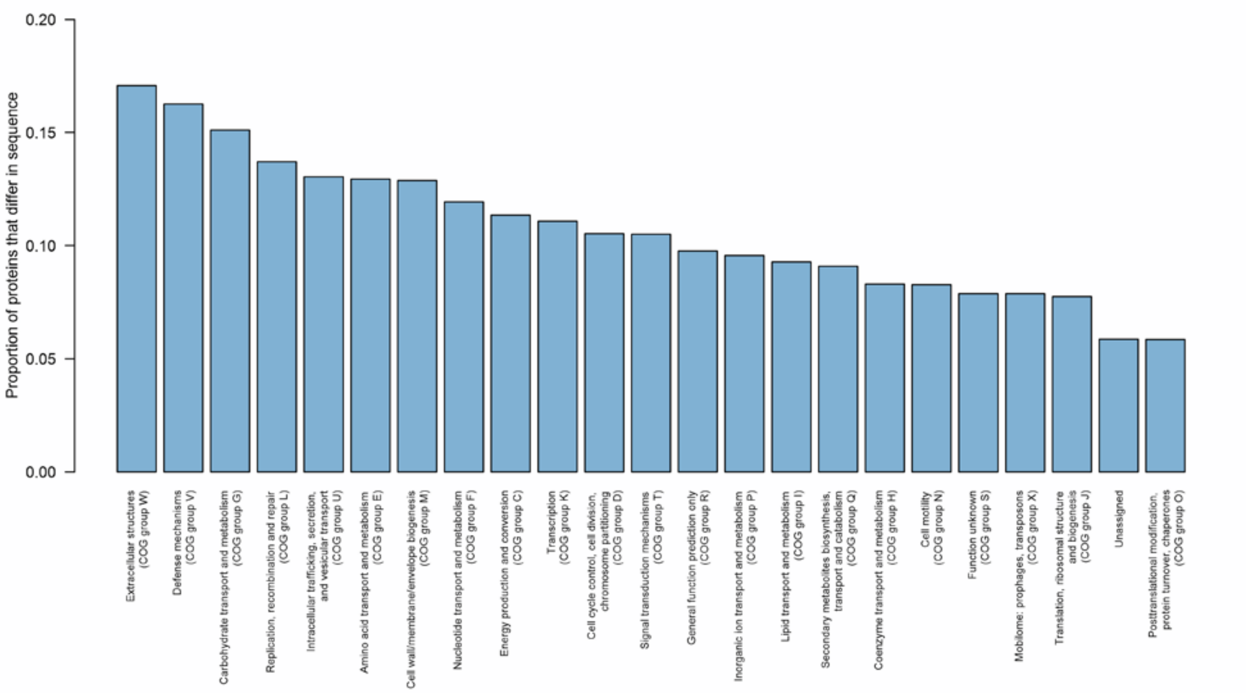

Technical Appendix Figure 9. Bar graph of the proportion of proteins that differ in sequence for each COG functional group.

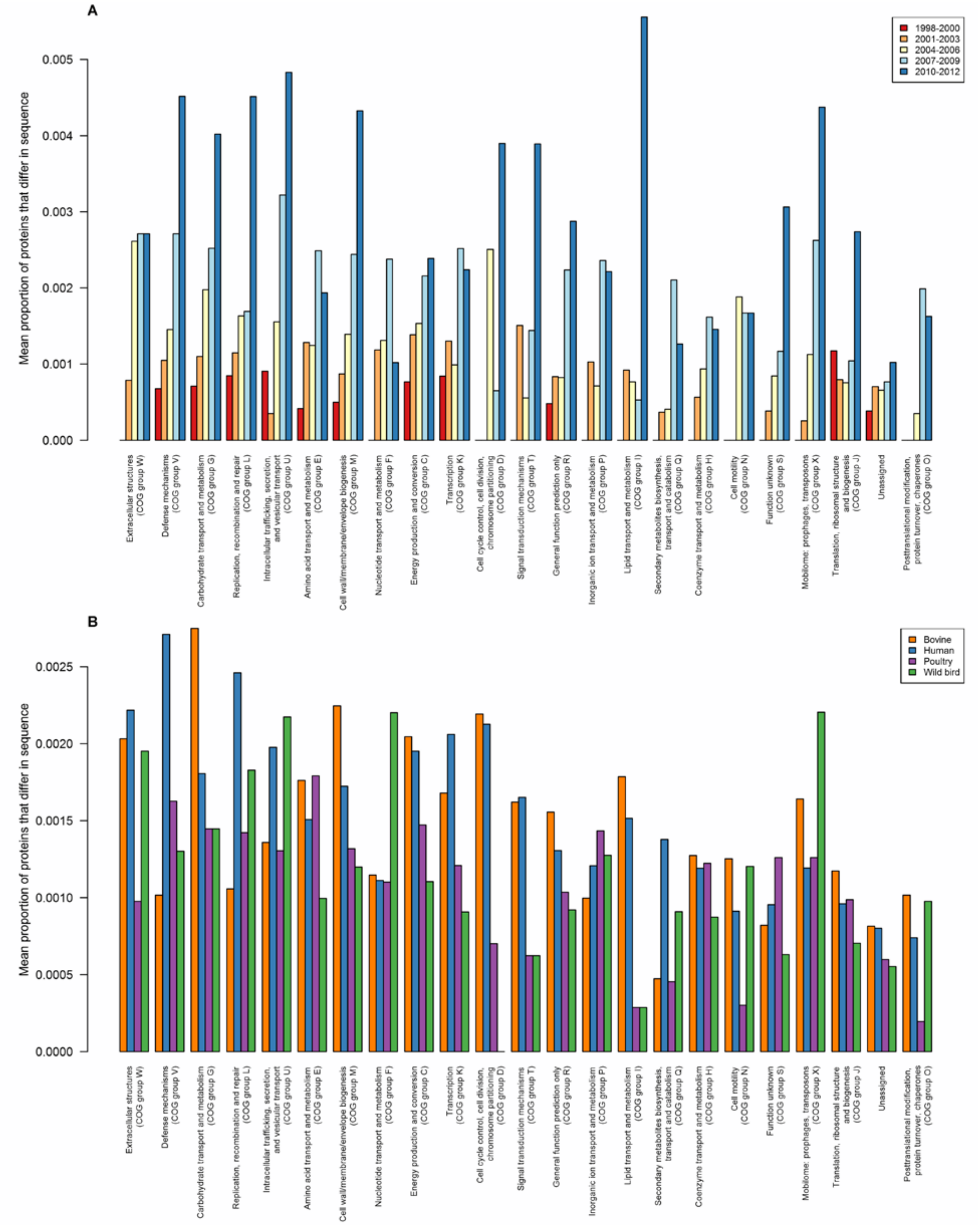

Technical Appendix Figure 10. Bar graph of the mean proportion of proteins that differ in sequence for each COG functional group within each time period (A) and source (B).

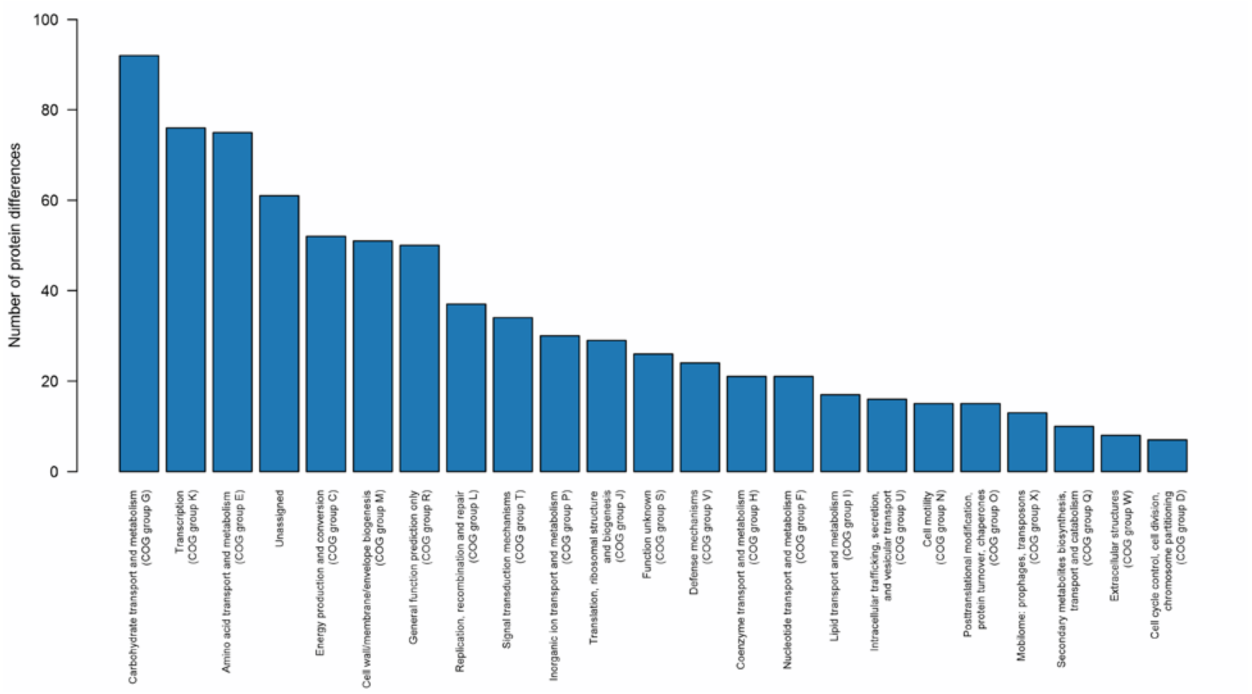

Technical Appendix Figure 11. Bar graph of the number of protein difference for each functional group shared by 107 DT160 isolates.

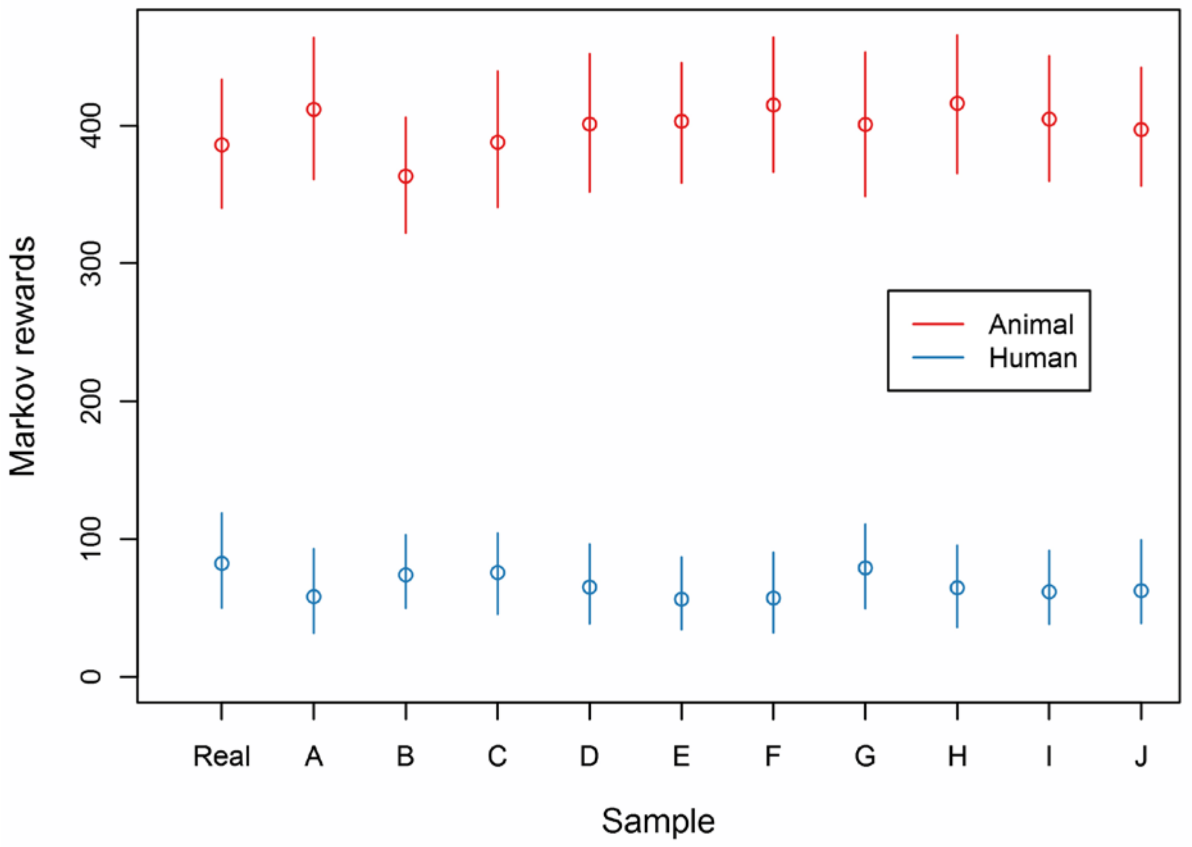

Technical Appendix Figure 12. Scatter plot of the number of animal (red) and human (blue) Markov rewards estimated for the real and ten randomly assigned (A-J) datasets. The circles represent the mean Markov reward value and the error bars represent the 95% HPD interval.

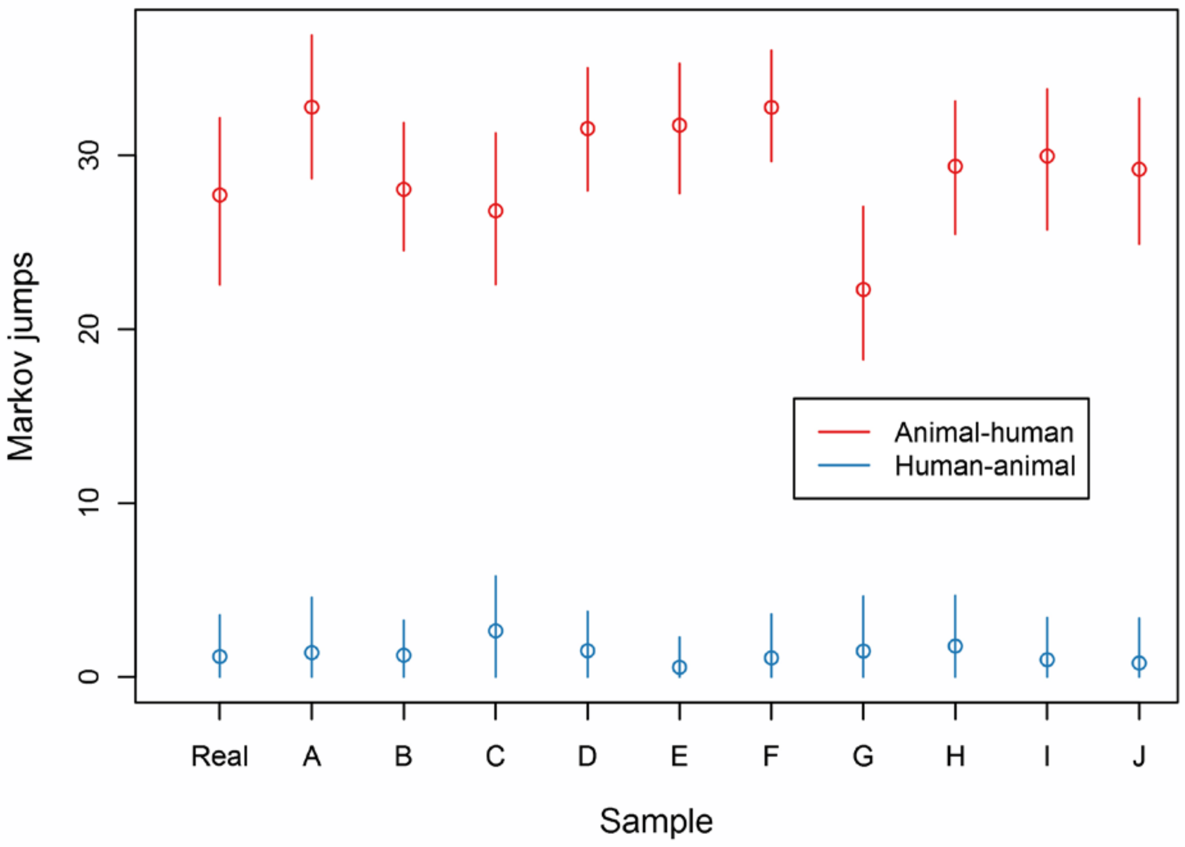

Technical Appendix Figure 13. Scatter plot of the number of animal-to-human (red) and human-to-animal (blue) Markov jumps estimated for the real and ten randomly assigned (A-J) datasets. The circles represent the mean Markov reward value and the error bars represent the 95% HPD interval.

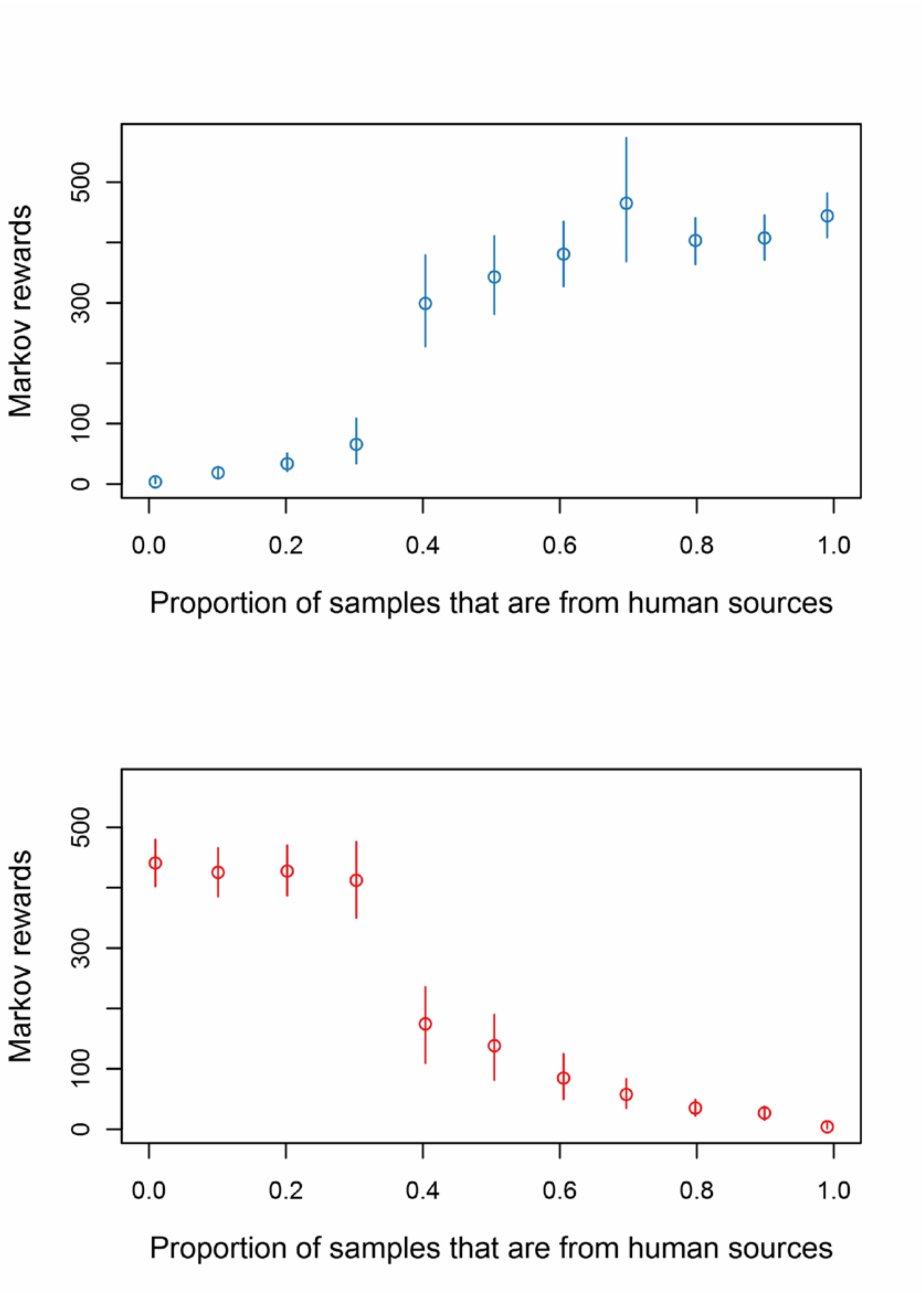

Technical Appendix Figure 14. Scatter plot of the number of animal (blue) and human (red) Markov rewards estimated versus the proportion of samples assigned as human. The circles represent the mean Markov reward value and the error bars represent the 95% HPD interval.

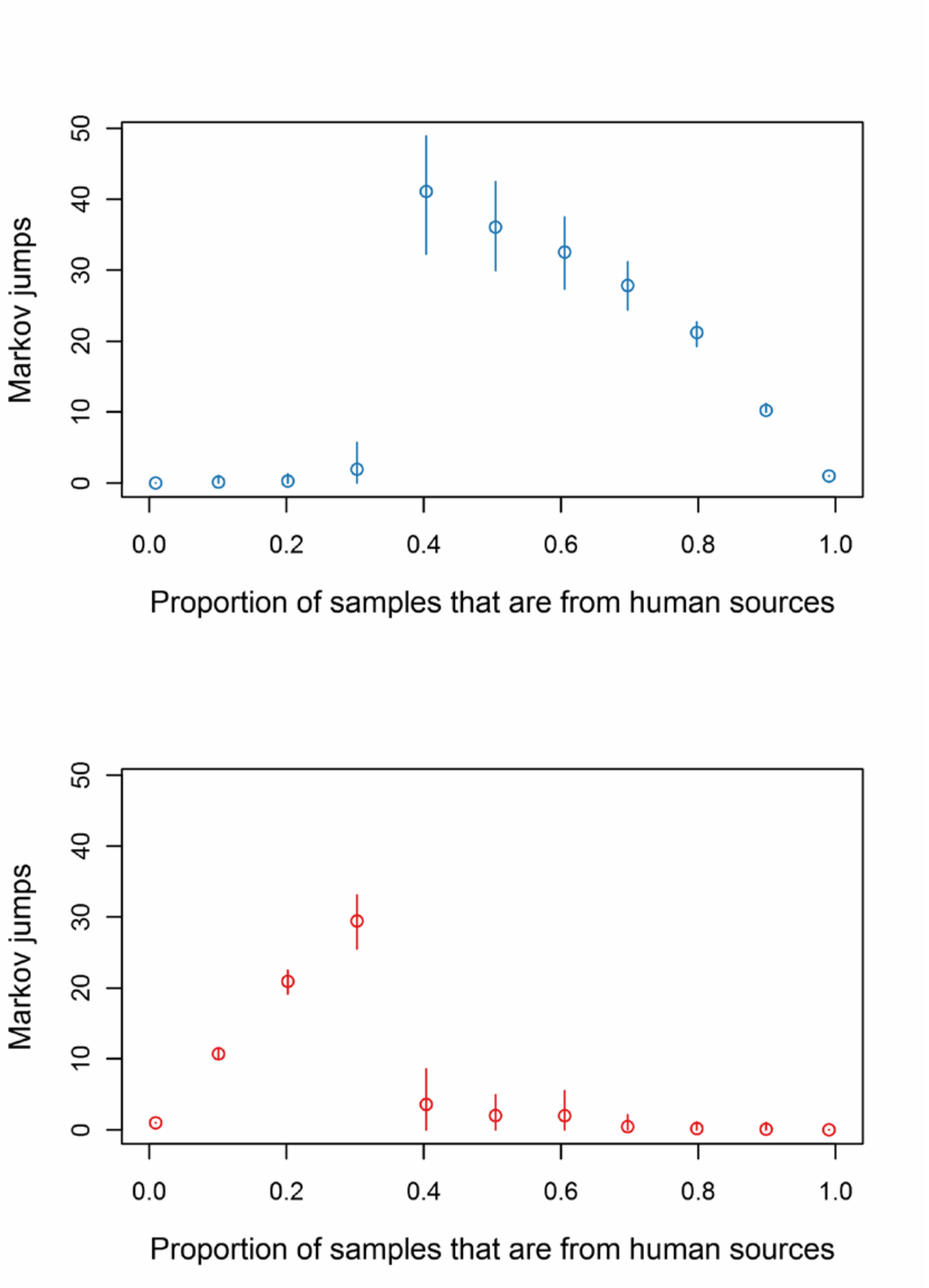

Technical Appendix Figure 15. Scatter plot of the number of animal-to-human (blue) and human-to-animal (red) Markov jumps versus the proportion of samples assigned as human. The circles represent the mean Markov jump value and the error bars represent the 95% HPD interval.

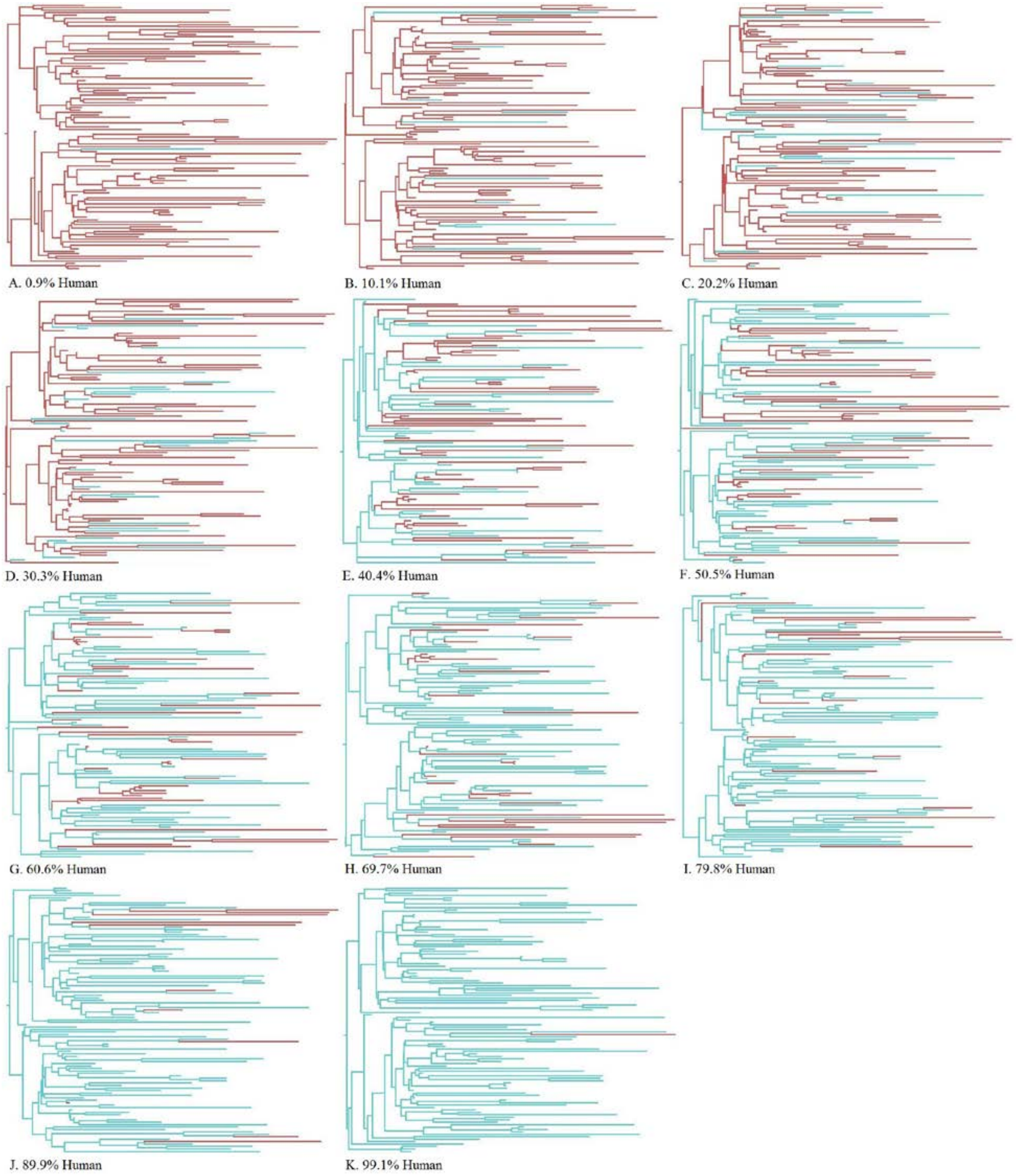

Technical Appendix Figure 16. Maximum clade credibility trees of 109 DT160 isolates placed through the discrete phylogeographic model, with different proportions of isolates assigned as human (blue) and animal (red).
